# Circadian Activity Rhythm in Early Relapsing–Remitting Multiple Sclerosis

**DOI:** 10.3390/jcm8122216

**Published:** 2019-12-15

**Authors:** Lorenzo Tonetti, Federico Camilli, Sara Giovagnoli, Vincenzo Natale, Alessandra Lugaresi

**Affiliations:** 1 Department of Psychology, University of Bologna, Viale Berti Pichat 5, 40127 Bologna, Italy; sara.giovagnoli@unibo.it (S.G.); vincenzo.natale@unibo.it (V.N.); 2 Dipartimento di Scienze Biomediche e Neuromotorie, Università di Bologna, Via Altura 3A, 40139 Bologna, Italy; federico.camilli@studio.unibo.it (F.C.); alessandra.lugaresi2@unibo.it (A.L.); 3 IRCCS Istituto delle Scienze Neurologiche di Bologna, UOSI Riabilitazione Sclerosi Multipla, Via Altura 3A, 40139 Bologna, Italy

**Keywords:** actigraphy, adult, circadian rhythm, motor activity, relapsing–remitting multiple sclerosis

## Abstract

While relapsing–remitting is the most prevalent course of multiple sclerosis, the prognostic/predictive markers of the worsening of symptomatology are still debated. With reference to other diseases, the study of the circadian activity rhythm, according to the theoretical framework of the two-process model of sleep regulation and applying functional linear modeling, proved to be useful to identify a possible marker. The usefulness of the study of circadian activity rhythm in multiple sclerosis is strengthened by recent findings indicating a potential involvement of circadian factors in the multifactorial etiopathology of the disorder. The aim of the present study was to verify whether circadian activity rhythm of early relapsing–remitting multiple sclerosis patients presents specific alterations, through functional linear modeling. Thirty-five relapsing–remitting multiple sclerosis patients (24 females; mean age ± SD = 31.51 ± 7.74) and 35 healthy controls (24 females; mean age ± SD = 31.29 ± 8.02) were enrolled. They wore an actigraph around the non-dominant wrist for one week. Relapsing–remitting multiple sclerosis patients showed a peak in motor activity around 5:00 a.m., higher than that of healthy controls. The timing of the peak in motor activity in the patients could be explained according to the hyperactive hypothalamus-pituitary-adrenal axis and higher cortisol awakening response reported in these patients.

## 1. Introduction 

Multiple sclerosis (MS) is a chronic autoimmune demyelinating disease of the central nervous system (CNS). The symptomatology of MS differs greatly according to the affected area of the CNS, with an unpredictable course at onset. Types of MS are relapsing–remitting MS (RRMS), secondary progressive MS (SPMS), and primary progressive MS (PPMS). RRMS is the most common form of MS at disease onset (85%–90% of patients) and can progress to SPMS with time. However, the predictive factors of this transition have not yet been clearly identified, although treatment can delay its onset [1].

From a chronobiological perspective, the investigation of the circadian activity rhythm (CAR) with reference to a different disorder (i.e., attention-deficit hyperactivity disorder) proved to be promising to detect a potential marker. Indeed, CAR, interpreted within the theoretical framework of the two-process model of sleep regulation [2] and examined through functional linear modeling (FLM) [3], allowed us to detect alterations in the homeostatic rather than circadian process of sleep regulation in adult attention-deficit hyperactivity disorder, with the identification of the lack of post-lunch dip as a possible trait marker [4]. Recent findings on genetic variability in ARNTL and CLOCK genes, known regulators of circadian rhythms, showed an association of specific genotypes to the risk of MS [5]. Furthermore, circadian rhythm disruptive factors, such as daylight saving time, showed an association to a higher prevalence of the disorder [6]. These findings point out a potential involvement of circadian factors in the multifactorial etiopathology of the disease. Nevertheless, the study of CAR in MS has received little attention. Indeed, to the best of our knowledge, only one study [7] has examined the CAR of MS patients (type of MS not specified), in blocks of five hours, but without a direct comparison with the CAR of healthy controls (HCs). Previous studies have explored the topic of circadian rhythm disorders in MS without examining the CAR. For example, Taphoorn and colleagues [8], using actigraphy, did not highlight a circadian disturbance in MS patients (half of the sample was in the relapsing–remitting phase and the other half in a progressive phase) assessed through the computing of non-parametric circadian variables. On the contrary, Najafi and colleagues [9] reported a higher prevalence of self-referred circadian rhythm sleep disorders in a sample of RRMS patients with a history of the disease of at least two years when compared to HCs.

The aim of the present study was to verify whether the CAR of early RRMS patients (less than 24 months from diagnosis) presents specific alterations, through FLM. With reference to the two-process model of sleep regulation, and consistently with the hyperactive hypothalamus-pituitary-adrenal (HPA) axis and higher release of cortisol found in early RRMS [10], an alteration of the circadian process can be expected.

Some novel aspects investigated in the present study can be highlighted. To the best of our knowledge, this is the first study to perform a detailed minute-by-minute analysis over the 24 h of CAR in MS. Moreover, we have analyzed the CAR of MS patients adopting, for the first time, the two-process model of sleep regulation [2] as the theoretical framework. Finally, we have examined a cohort of patients in the early phase of the disease, where lesion load and disability are lower, therefore decreasing the impact of confounding factors.

## 2. Materials and Methods

### 2.1. Participants

Thirty-five RRMS patients (24 females and 11 males; mean age: 31.51 ± 7.74 years) were enrolled at the referral center for the diagnosis and treatment of MS at the “Istituto di Ricerca e Cura a Carattere Scientifico (IRCCS)”–“Istituto delle Scienze Neurologiche di Bologna (ISNB)” (Bologna, Italy). The inclusion criteria were the following: age between 18 and 65 years; diagnosis of RRMS; less than 24 months from the diagnosis; disease modifying treatment (DMT) for more than 6 months, and no relapse or steroid treatment in the last month; no anamnestic evidence of comorbidity with severe/acute diseases or psychiatric disorders; stable symptomatic therapy in the two weeks preceding the enrolment; absence of disabilities interfering with motor activity; absence of diagnosed sleep disorders; no engagement in shift work with night shifts during the observation period. With reference to ongoing pharmacological treatment at the time of enrollment in the present study, 28.57% (*n* = 10) of patients were taking glatiramer acetate, 25.71% (*n* = 9) peginterferon beta-1a, 20% (*n* = 7) dimethyl fumarate, 14.29% (*n* = 5) subcutaneous or intramuscular interferon beta-1a, 5.71% (*n* = 2) teriflunomide, 2.86% (*n* = 1) fingolimod, and 2.86% (*n* = 1) natalizumab.

Thirty-five HCs (24 females and 11 males; mean age: 31.29 ± 8.02 years) were jointly enrolled at the center for MS of the IRCCS–ISNB and the Laboratory of Applied Chronopsychology at the University of Bologna. The inclusion criteria were: absence of possible undiagnosed sleep disorders; no anamnestic evidence of psychiatric disorders or acute or severe illnesses; no use of psychoactive drugs; absence of disabilities limiting motor activity; no shift work with night shifts. The inclusion criteria were verified through a semi-structured interview during which potential participants filled in the Sleep Disorders Questionnaire [11].

RRMS patients and HCs were matched for gender (χ^2^_1_ = 0; *p* = 1) and age (t_68_ = 0.12; *p* = 0.90).

### 2.2. Actigraphy

The actigraph model Micro Motionlogger Watch (Ambulatory Monitoring, Inc., Ardsley, NY, USA) was used. The hardware was composed of a triaxal accelerometer, with a sensitivity of 0.01 g, filters at 2–3 Hz, and a sampling frequency of 32 Hz. The actigraphs were initialized in zero crossing mode through the Motionlogger Watchware software (Ambulatory Monitoring, Inc., Ardsley, NY, USA) in order to acquire motor activity data in 1-min epochs. Motor activity data can also be transformed into dichotomous information about sleep and wake according to validated algorithms [12,13].

### 2.3. Analysis of Sleep/Wake Behavior

The Action W 2.7.1150 software (Ambulatory Monitoring, Inc., Ardsley, NY, USA) was used in order to describe the sleep/wake behavior of participants.

The following actigraphic sleep parameters were examined: bedtime (BT), the time of day when participants went to bed trying to fall asleep; get-up time (GUT), the time of day when participants got out of bed; time in bed (TIB), the interval in minutes between BT and GUT; midpoint of sleep (MS), the time of day that splits the TIB in half; sleep motor activity (SMA), the motor activity counts in 1-min epochs during the assumed sleep time; sleep onset latency (SOL), the interval between BT and sleep onset (SO), defined as the first epoch of a block of 20 consecutive sleep epochs with no more than one epoch of wake; total sleep time (TST), the sum in minutes of the sleep epochs between SO and GUT; wake after sleep onset (WASO), the sum in minutes of the wake epochs between SO and GUT; sleep efficiency (SE), the ratio between TST and TIB multiplied by 100; awakenings (AWK), the number of awakenings; awakenings lasting more than 5 min (AWK > 5).

We also examined the following actigraphic wake parameters: diurnal motor activity (DMA), the motor activity counts in 1-min epochs during the assumed wake period; diurnal total sleep time (DTST), the sum in minutes of sleep epochs between GUT and BT; diurnal sleep episodes (NAP), the number of sleep episodes within the interval defined by GUT and BT; duration of the longest sleep episode (NAPD).

The means of the previous sleep and wake actigraphic parameters were computed for working days only.

### 2.4. Circadian Activity Profile

The Action 4 software (Ambulatory Monitoring, Inc., Ardsley, NY, USA) was used in order to extract the minute-by-minute raw motor activity over 24 h for each working day. Then, for each participant, the mean of all working days was computed, allowing us to describe the raw circadian activity profile.

### 2.5. Procedure

Both RRMS patients and HCs were requested to wear the actigraph around the non-dominant wrist 24 h per day for seven consecutive days [14,15]. Moreover, they were instructed to push the event-marker button on the top of the actigraph in order to signal BT and GUT. When they failed to push the event-marker button, the scorer of actigraphic records referred to the replies to the questions on BT and GUT reported in the Core Consensus Sleep Diary [16] filled in daily by the participants.

The Bologna-Imola Ethics Committee (general protocol number 0122151 of 18 October 2017; study number 17113) approved the research protocol and each participant provided written informed consent prior to the inclusion in the study.

### 2.6. Statistical Analyses

We compared the sleep/wake behavior of RRMS patients and HCs through a set of independent samples t-test with the group (RRMS patients and HCs) as the independent variable and each actigraphic parameter as the dependent variable. Because multiple comparisons were performed, the Bonferroni correction (alpha level divided by the number of comparisons, i.e., 0.05/15) was applied, considering as significant only the *p* values less than 0.003.

The CAR of RRMS patients and HCs was compared through the FLM [3]. Using the R “Actigraphy” package, the mean-raw circadian activity profile of each group was firstly transformed into a functional form, through a Fourier expansion model (9 Fourier basis permutation) fitted at a periodicity of 24 h, and then compared with each other through a set of non-parametric permutation F-test, allowing us to point out the times of day with significant differences in motor activity between the groups.

## 3. Results

The comparison between RRMS patients and HCs with reference to actigraphic sleep/wake measures is shown in Table 1. As regards the sleep period, four significant differences were observed, with RRMS patients presenting higher SMA, AWK > 5, and SOL, as well as lower SE than HCs. With reference to the wake period, no significant differences between groups were observed.

**Figure 1:**
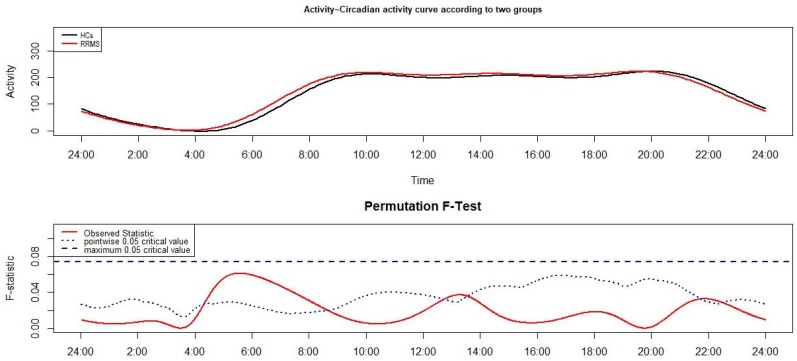
Functional linear modeling (FLM) applied to the comparison of the circadian activity rhythm (CAR) between relapsing–remitting multiple sclerosis (RRMS) patients and healthy controls (HCs). The functional forms of the mean CAR of groups are reported in the upper panel of the figure and the results of the non-parametric permutation F-Test in the lower panel.

The results of the FLM analysis are reported in Figure 1. In the upper panel are reported the CAR of RRMS patients and HCs, and the results of the non-parametric permutation F-test are reported in the lower panel. Differences between groups are detected when the observed statistic (i.e., the red solid line) is above the test of significance with the α level set at 0.05. The blue dashed line represents the global test of significance, while the dotted line represents the point-wise test of significance. Although the global test is preferred because it represents the most conservative level of significance, the point-wise test of significance, even if less conservative, is a useful threshold to detect relevant differences in motor activity [3]. Observing Figure 1, it is possible to note that RRMS patients show a peak in motor activity, around 5:00 a.m., higher than that of HCs.

## 4. Discussion

The results shown in Table 1 point out that RRMS patients and HCs present similar sleep timing (BT, GUT, TIB, and MS) and sleep quantity (TST). However, RRMS patients present an impaired sleep quality in terms of a reduced SE (main marker of sleep quality) and a higher SMA, AWK > 5, and SOL. Although we observed this pattern of differences between groups, we should acknowledge that, roughly comparing the mean values of actigraphic sleep parameters with the clinical cut-off scores proposed by Natale and colleagues [17] for this specific actigraphic device, only SOL and AWK>5 are slightly above such cut-off values. These data seem to point out a slight difficulty in the onset and maintenance of sleep that is not highly significant, possibly because patients are in the early phase of the disease and do not present severe symptoms known to interfere with sleep, such as sphincter disturbances, painful sensory symptoms, motor spasms, or restless legs syndrome.

Since this is the first study that has examined in detail the CAR of early RRMS patients, through FLM, within the theoretical framework of the two-process model of sleep regulation [2], no benchmark is currently available for comparison. However, the peak in motor activity around 5:00 a.m. observed in RRMS patients compared to HCs can be interpreted within the framework of a hyperactive HPA axis, previously reported in MS patients [18], and the resulting increase of the release of cortisol [19]. We have to acknowledge that this is just a correlative and descriptive speculation, which should be taken with caution. The functioning of the HPA axis can be conveniently assessed through the measuring of the cortisol awakening response. There was a marked increase in the release of cortisol 15–20 min after the morning awakening, which was preceded by an increase of cortisol secretion in the second part of the night, when people were sleeping [20]. Due to the ecologic nature of our study, measurements of cortisol levels were not obtained. It is however suggestive that a previous study reported a higher cortisol awakening response in patients with early RRSM compared to HCs [10], providing data supportive of the results observed in our work and the hypothesis formulated. Furthermore, this interpretation is in line with the hypothesis of the involvement of circadian disruptive factors in an extra effort of the suprachiasmatic nucleus (the anatomical location of the biological clock) to entrain the circadian rhythms that would affect the HPA axis, leading to a desynchronization of the circadian clock and an abnormal immune response [6], which is known to play a critical role in the etiopathogenesis of MS. Such hypothesis is supported by the detection of a higher prevalence of MS in countries where daylight saving time, a circadian disruptive factor, is adopted [6]. Our hypothesis is also supported by the evidence of a higher risk of developing MS in those who started shift working, another circadian disruptive factor, before the age of 20 [21]. It is known that the circadian timing system completes its maturation at the end of adolescence, around 20 years of age [22], potentially explaining the major disruptive role of shift work in adolescents with a still developing circadian timing system. Furthermore, data from animal models also seem to strengthen the role of circadian alterations in MS. Indeed, it has been shown [23] that in a mouse model of MS (i.e., mice with experimental autoimmune encephalomyelitis, EAE) both the expression of the clock gene and hormonal rhythms were altered. In particular, EAE mice presented elevated levels of corticosterone. Finally, according to the theoretical framework of the two-process model of sleep regulation, the peculiar hyperactivity in RRMS patients seems to confirm a possible alteration in the circadian process.

A still open question pertains to the generalizability of the present findings to other demyelinating disorders or their specificity for early RRMS. As regards CAR, we are not aware of previous studies on other demyelinating disorders. To the best of our knowledge, little has been published on other demyelinating inflammatory diseases other than case reports regarding specific demyelinating lesions in areas relevant to sleep. A recent work by Barzegar and colleagues [24] investigated the sleep complaints of persons with neuromyelitis optica compared to healthy controls through questionnaires. Many confounding factors were present and correlated with poor quality of sleep. In particular, it is known that in neuromyelitis optica spectrum disorder (NMOSD), lesions in the hypothalamus and thalamus can be present and interfere with sleep, sometimes causing symptomatic narcolepsy [25] (to have a better overview on sleep disturbances in relation to the site of lesions, please refer to a recent review by Foschi and colleagues [26]). Therefore, the point, in our opinion, remains open and is worthwhile to be explored in future studies on a larger number of patients, to enable a meaningful subgroup analysis.

The results of our study might have been affected by the ongoing pharmacological treatment of RRMS patients. The use of interferon beta, glatiramer acetate [27], and dimethyl fumarate (personal observation) might have caused, even in the steady state phase, a subtle disruption of sleep, although there are no available data on the effects of these drugs on sleep in the drugs’ technical sheets. A recent review on the subject [28] reported possible negative effects caused by glatiramer acetate and interferon beta, whereas data on first line oral drugs (dimethyl fumarate and teriflunomide) are not yet available.

The present study is not free from limitations, due to the relatively small size of the early RRMS patient sample, as well as the lack of assessment of cortisol secretory activity and possible confounding factors such as mild symptoms of anxiety and depression through available standardized questionnaires; fatigue, a possible consequence of poor sleep, would also merit investigation. Future studies should be conducted on larger groups of patients and controls, collecting data on possible confounding factors. To measure cortisol secretion, in order to better understand the possible role played by cortisol in the modulation of CAR, would be useful but impractical in an outpatient cohort. Furthermore, it would be extremely interesting to perform a prospective study with a pre-planned periodic follow-up, with the aim of assessing whether the peculiar hyperactivity in early RRMS patients may occur in relation with different parameters such as persistent disease activity, response to treatment, and different treatment options, and with the correlation to different disease courses/activity, therefore representing a predictive marker of disease activity or progression.

## Figures and Tables

**Table 1:** Means and standard deviations of actigraphic sleep/wake measures in early relapsing–remitting multiple sclerosis (RRMS) patients and healthy controls (HCs). Statistics are also reported, with significant effects in italic.

Actigraphic Measure	RRMS Patients	HCs	Statistics	
			t_(68)_	*p* ^a^
**Sleep**				
BT	23:39 ± 1:04	23:58 ± 0:55	−1.33	0.19
GUT	07:08 ± 1:05	07:16 ± 0:41	−0.65	0.52
TIB	449.21 ± 56.86	439.37 ± 56.09	0.73	0.47
MS	03:23 ± 0:58	03:37 ± 0:40	−1.14	0.26
SMA	13.52 ± 5.13	10.08 ± 2.26	3.62	<0.001
SOL	15.20 ± 11.49	8.45 ± 4.24	3.26	<0.003
TST	413.61 ± 52.68	419.78 ± 54.02	−0.48	0.63
WASO	20.91 ± 19.57	10.22 ± 6.21	3.08	0.003
SE	92.14 ± 5.11	95.57 ± 1.53	−3.80	<0.001
AWK	9.72 ± 4.76	7.27 ± 2.46	2.70	0.009
AWK > 5	2.23 ± 1.64	1.21 ± 0.50	3.52	<0.001
**Wake**				
DMA	209.28 ± 21.08	202.74 ± 21.70	1.28	0.21
DTST	28.18 ± 32.92	22.63 ± 18.15	0.87	0.38
NAP	3.34 ± 2.88	3.38 ± 2.37	−0.08	0.94
NAPD	15 ± 16.33	11.64 ± 8.93	1.07	0.29

BT = bedtime (h:min); GUT = get-up time (h:min); TIB = time in bed (min); MS = midpoint of sleep (h:min); SMA = sleep motor activity (counts); SOL = sleep onset latency (min); TST = total sleep time (min); WASO = wake after sleep onset (min); SE = sleep efficiency (%); AWK = awakenings (number); AWK>5 = awakenings lasting more than 5 min (number); DMA = diurnal motor activity (counts); DTST = diurnal total sleep time (min); NAP = diurnal sleep episodes (number); NAPD = duration of the longest sleep episode (min). ^a^ Since multiple comparisons were performed, the Bonferroni correction was applied, leading us to consider as significant *p* values less than 0.003.
